# Increasing STEM Skills, Knowledge and Interest Among Diverse Students: Results from an Intensive Summer Research Program at the University of California, San Francisco

**DOI:** 10.1007/s10755-024-09701-z

**Published:** 2024-02-23

**Authors:** Gino Galvez, David W. Killilea, Sharla Berry, Vasanthy Narayanaswami, Ellen B. Fung

**Affiliations:** 1 Department of Psychology, California State University, Long Beach, CA 90840 USA; 2 Office of Research, University of California, San Francisco, CA USA; 3 Department of Educational Leadership, California State University, Long Beach, CA USA; 4 Department of Chemistry and Biochemistry, California State University, Long Beach, CA USA; 5 Division of Hematology, Department of Pediatrics, University of California, San Francisco, CA USA

**Keywords:** Underrepresented minority students, STEM education, Summer research program, Research skills

## Abstract

This study evaluates the effectiveness of the UCSF Summer Student Research Program (SSRP) in enhancing research-related skills, academic outcomes, and post-baccalaureate aspirations of underrepresented minority (URM) and non-URM undergraduate students in biomedical sciences and STEM fields. The SSRP, spanning 9 weeks, provides immersive research experiences, structured mentorship, trainings, seminars, and STEM education. Pre- and post-program survey data from eight cohorts (N = 315) were analyzed using paired-sample *t*-tests, MANOVA, and content analysis. Results demonstrate significant gains in critical thinking skills, research abilities, science identity, applied science skills, and readiness for a research career. Notably, participants exhibited improvements in understanding the research process, scientific thinking, science writing, and problem-solving. URM and non-URM students experienced similar gains, highlighting the program’s inclusivity. The SSRP also positively influenced students’ postgraduate aspirations. Some participants expressed heightened interest in pursuing Master of Arts, Ph.D., and M.D. degrees, indicating increased clarity and motivation towards advanced education and research careers. Furthermore, 87% of participants expressed a high likelihood of engaging in future research endeavors, underscoring the program’s sustained impact on research interest. This study underscores the transformative potential of a well-structured, intensive summer research program in significantly enhancing academic outcomes for URM and non-URM students alike. These findings align with the persistence framework, emphasizing the importance of early research experiences, active learning, and learning communities in fostering student success. The SSRP’s effectiveness in improving research skills and post-baccalaureate aspirations suggests its potential in diversifying the STEM fields, biomedical sciences and health-related professions.

## Introduction

Over the last two decades, there has been an increase in national calls for initiatives and educational enrichment programs to increase and retain the number of undergraduate students in the science fields to address the shortage of individuals in the biomedical sciences and science, technology, engineering and mathematics (STEM) workforce (Pender et al., 2010; President’s Council of Advisors on Science and Technology [PCAST], 2012). However, degree attainment in the STEM fields across groups suggests that underrepresented minority (URM; African American, Hispanic or Latino/Latina, American Indian, and Alaska Natives) college students are disproportionately leaving the sciences or leaving institutions without a degree when compared to White and Asian students (Estrada et al., 2016; PCAST, 2012). Moreover, the gaps in participation rates between URMs and White and Asian students further widen at the graduate and professional school levels (Sullivan Commission, 2004). Addressing the shortage in the diversity of individuals in the STEM fields is a critical issue that impacts the future of our nation’s health and economic prosperity.

While a broad set of interventions and institutional changes are needed to address the systemic educational disparities (Chang et al., 2008; Estrada et al., 2016), evidence indicates that research experiences are effective educational strategies to increase students’ pursuit and persistence in STEM fields (Foertsch et al., 2000; Lopatto, 2004). Moreover, literature indicates that mentored undergraduate research experiences may be an effective method of participation in the STEM fields among URM students (Jones et al., 2010; Lopatto, 2004, 2007; Nagda et al., 1998;). Conducting research has been shown to enhance student learning experiences, boost interest in STEM careers, encourage pursuit of graduate education in the STEM fields, increase engagement and persistence in STEM degree programs (Pender et al., 2010).

Graham and colleagues (2013) proposed an evidenced-based persistence framework that integrates psychology and education research on motivation, engagement, and self-efficacy to increase persistence among students (i.e., student agency) in the STEM fields. The framework identifies three key areas associated with the determinants of learning and professional identification, namely (1) early research experiences, (2) active learning, and (3) membership in learning communities. The data indicates that students who engage in research in the first two years of college are more likely to persist in STEM majors (Nagda et al., 1998). This is especially important given that most students conduct research in the latter years of college which is after the critical period of attrition in STEM majors in the freshman year (Russell et al., 2007). Active learning (e.g., problem solving) has been shown to increase retention as it improves understanding and retention of concepts and information. Furthermore, active learning helps student identify as scientists because they actively participate in scientific thinking with peers thus creating scientific communities. Lastly, membership in STEM learning communities provide structure that allow students to work with and learn from each other. Taken together, this research shows that strengthening undergraduate students’ self-efficacy and science identity increases student retention and persistence in the biomedical science and STEM fields (Chemers et al., 2011; Estrada et al., 2011).

While we know that intensive research programs positively impact STEM students’ persistence and retention in the field, there is a lack of empirical data on the impact of summer research programs both generally, and specifically on racial/ethnic minorities. As we seek to understand the types of programs that benefit diverse learners, we need to know more about the impact of different formats, including short term intensive research programs, on student success. This study explores one innovative format, the 9-week summer intensive program.

Undergraduate summer research experiences typically offer opportunities for students to conduct research at a host institution over the summer months and primarily work with a mentor on a research project. Undergraduate summer research experiences are frequently incorporated into larger federally-funded research training initiatives (Urizar et al., 2017). Noted benefits of summer research experiences include increasing skills in research, interest in and preparation for graduate school, illuminating potential career opportunities in the STEM workforce, and enhancing the overall college experience (Hathaway et al., 2002; Lopatto, 2003). Undergraduate STEM students have also reported being more motivated and confident as a result of summer research participation (Seymour et al., 2004). Summer programs that provide a combination of opportunities for early research experiences, active learning, and membership in learning communities (i.e., hallmarks of a “persistence” framework) may be poised to increase research-related skills and knowledge, self-efficacy, science identity and persistence among students in the STEM fields.

Despite what we know about summer research programs, there are still some gaps in the research and data is limited (Pender et al., 2010). For example, there is a lack of well-designed studies and empirical data using statistical analyses that explore the impact of summer research programs both generally, and specifically on racial/ethnic minorities (Pender et al., 2010; Seymour et al., 2004). This study begins to fill this gap.

In an effort to promote diversity in the biomedical sciences and health-related professions, the University of California San Francisco (UCSF) Summer Student Research Program (SSRP) was designed to provide opportunities for students to immerse themselves in research during the summer. The purpose of this study is to examine gains and outcomes associated with the participants’ completion of the summer program. The research questions addressed in this study are:

1. Does participation in the SSRP improve research-related skills and abilities?
2. Are there differences in the outcomes between URM and non-URM students who participated in the SSRP?
3. What is the impact on interest in research, research career, and future research endeavors for students who participated in the SSRP?

## Study Context

UCSF Benioff Children’s Hospital Oakland (BCH-Oakland) is a non-profit, public-benefit children’s hospital and is located in one of the most diverse cities in the U.S. BCH-Oakland’s long-standing commitment to providing educational opportunities (i.e., training and mentoring activities) for students and professionals combined with state-of-the-art basic, clinical, and translational opportunities has made it a fertile environment for fostering interest and training in biomedical research. Despite changes in custodianship since its inception, the SSRP has persisted without interruption for 42 years. The program provides a structured mentored research training experience for students from diverse backgrounds (i.e., low-income, first-generation college student), racial and ethnic minorities, women, and others who are underrepresented in biomedical research, health-related professions (e.g., medicine, dentistry) and STEM fields.

The SSRP’s 9-week structured curriculum provides students with an immersive experience in a research community in which they acquire firsthand knowledge of various research types, understand the scientific process, and cultivate skills in the laboratory, communication, and analysis. Participants conduct their own research projects within a laboratory or clinical setting under the mentorship of an experienced investigator; attend regular structured seminars, discussion groups, carefully selected didactic presentations intended to impart a strong foundation in biomedical research and provide practical advice for pursuing research and health-related careers. It is intended for participants to develop critical thinking skills; gain a robust introduction to study design, scientific methods, and data analysis; learn about research ethics; and develop a realistic understanding of conducting research. The program culminates in a public presentation of their research findings at the end-of-summer symposium.

Employing an apprenticeship model, the program aims to promote interest in science by offering high quality research internships in a welcoming, hands-on, real-world setting. Mentors play a key role guiding the participant through the program through structured activities and meetings. Participants follow the procedures and schedule laid out by their mentors and many work with other undergraduate and graduate students, postdoctoral researchers, and clinical staff. Mentors are expected to assist students in becoming familiar with the scientific literature in their specific research area, identify and properly formulate a research question, design a study methodology to test their research question, collect, analyze, and interpret data, and present results.

Recruitment extensively reaches out to high schools and community colleges in the Bay Area, as well as local programs and universities, with a focus on minority-serving institutions. Application materials (e.g., personal statements, transcripts, letters of recommendation) are required and undergo a holistic assessment which emphasizes identifying promising students rather than relying solely on academic credentials. General criteria include high school juniors or seniors completing at least one year of math and biology, or undergraduates currently enrolled in a U.S. academic institution with an interest in a STEM field.

## Methodology

### Data Sources

This study utilized data from the ongoing SSRP evaluation, retrospectively analyzing survey data from 2012 to 2019 years in which the program was consistent in design (i.e., in-person, didactic, structured curriculum; see Fung et al. (2021) for results of the virtual/hybrid program during the COVID-19 pandemic). As part of the evaluation, surveys were administered to participants to obtain pre- and post-program data on demographic and outcome variables. The survey items assessed student perceptions of their: (a) educational aspirations, (b) motivation to participate in summer research program, (c) assessment of their research experience, (d) evaluation of their mentor, and (e) assessment of research skills and abilities. Survey questions were adapted for a pre-post design from validated measures such as the Summer Undergraduate Research Experience (SURE; Lopatto, 2007) and the Undergraduate Research Student Self-Assessment (URSSA; Weston & Laursen, 2015). For most items, participants rated their agreement with statements using a 5-point Likert scale ranging from “strongly disagree” to “strongly agree.” Both the pre- and post-program surveys consisted of a subset of 20 items (i.e., research-related abilities, skills, and gains) which allowed for comparisons to be made between the time points.

### Procedures

Utilizing Qualtrics, data from participants were collected at two time points: (1) before starting the SSRP (pre-survey) and (2) after completing the SSRP (post-survey) across eight separate cohorts. The online surveys were made available the week prior to the start of the summer program and the week following their completion of the program. Participants received emails from the program coordinators, which contained an internet link to the online survey as well as a brief description and purpose of the voluntary survey. The study was approved by the Internal Review Board at California State University Long Beach (1028029-2).

Databases were cleaned and analyzed using IBM SPSS Statistics (Version 27). Descriptive statistics are reported as means (with standard deviations) and frequencies are reported as percentages of valid data and exclude missing data. Paired-samples *t*-tests were conducted to compare mean differences on 20 identical pre- and post-survey items (i.e., research-related abilities, skills, and gains). Holm’s procedure was applied to all 20 paired-sample *t*-test *p* values to control the family-wise error rate (Aickin & Gensler, 1996). Effect sizes were calculated using Cohen’s d to assess the magnitude of the difference between the means. The interpretation of the reported effect size was according to magnitude criteria for the behavioral sciences (i.e., small = 0.20, medium = 0.50, and large = 0.80; Cohen, 1988). A MANOVA was conducted using the Multivariate General Linear Model (GLM) to determine whether differences exist between URM and non-URM participants on post-survey items (i.e., research-related abilities, skills, and gains). Holm’s procedure was applied to all follow-up *F*-test *p* values to control the family-wise error rate (Aickin & Gensler, 1996). Effect sizes were calculated using partial eta-squared to assess the magnitude of the difference between the means. The interpretation of the reported effect size was according to magnitude criteria for the behavioral sciences (i.e., small = 0.01, medium = 0.06, and large = 0.14; Cohen, 1988). Finally, text-based comments from open-ended items on the post-survey were analyzed using a content analysis approach. Content analysis is a method used to quantify and interpret meaning from the text data through the systematic process of coding and identifying the primary themes or patterns (Patton, 1990). All comments were imported into the NVivo (Version 12) for coding and analysis.

## Results

### Demographics

A total of 315 students participated in the pre- and post-program surveys across eight cohorts (mean number of students per cohort = 39.4, *SD* = 7.8). The mean response rate for the pre-survey was 93.5% (*SD* = 6.0) and the post-survey was 83.2% (*SD* = 11.7). The mean age of the participants was 18.8 years (*SD* = 2.2). Among them, 62.9% identified as female, 47.3% belonged to underrepresented minority (URM) groups, while 52.7% identified as non-URM groups. See Table 1 for additional demographic results.

**Table 1:** Participant demographic characteristics

Characteristic	% (n)
*Self-reported Gender*	
Female Male Trans	62.9 (198) 36.8 (116) 0.3 (1)
*Grade Level*	
High School Junior High School Senior Freshman Sophomore Junior Senior	9.5 (30) 23.8 (75) 11.4 (36) 14.6 (46) 24.4 (77) 16.2 (51)
*Underrepresented Minority (URM)*	47.3 (147)
Self-reported Ethnicity	
African American/Black Asian Hispanic/Latino Middle Eastern Native American Pacific Islander White More than category selected Other	14.5 (45) 24.8 (77) 30.9 (96) 6.4 (20) 1.0 (3) 1.0 (3) 14.1 (44) 3.5 (11) 3.9 (12)
*Academic Majors*	
Biology Biochemistry Chemistry Computer Science Engineering Neurobiology Physics Psychology Other: Humanities Other: Natural Sciences Other: Social Sciences Undecided	44.6 (136) 9.5 (29) 2.0 (6) 0.3 (1) 3.9 (12) 8.5 (26) 0.3 (1) 3.0 (9) 2.6 (8) 2.6 (8) 5.9 (18) 16.7 (51)

*Note* URM refer to African American/Black, Hispanic/ Latino, and American Indian or Alaska Native (NSF, 2019). The range of gender categories was expanded in 2017

### Participants’ Terminal Degree Intention

Participants were asked about their future plans over the next few years for continuing their education at pre- and post-program. The percentages and frequencies are shown in Table 2. In general, an increase in intention for Ph.D. in science-related field and M.D. was observed at post-program. In contrast, decrease in intention for jointly pursuing an M.D./Ph.D. program was observed post-program.

**Table 2:** Participants’ Terminal Degree Intention at Pre- and Post-program

Terminal Degree	Pre	Post
	% (n)	% (n)
Master’s in science-related field Master’s in a non-science-related field Ph.D. in science-related field M.D. M.D./Ph.D. J.D. or other professional degree Not considered options for post-undergraduate education Plan not to pursue post-undergraduate education Other	14.9 (45) 0.7 (2) 11.6 (35) 47.2 (143) 13.5 (41) 0.7 (2) 6.6 (20) 1.3 (4) 3.6 (11)	15.6 (46) 0.3 (1) 15.0 (44) 56.8 (167) 2.0 (6) 2.7 (8) 5.8 (17) 0.3 (1) 1.4 (4)

*Note* Missing data (pre, n = 12; post, n = 21)

### Motivation to Participate in the Summer Research Program

Ten questions were asked on the pre-survey to determine participants’ motivation for participating in the summer research program. Overall, means ranged from 3.6 to 4.8 with most indicating that participants tended to “agree” or “strongly agree” with each statement. To compare responses between URM and non-URM participants, independent samples *t*-tests were conducted. Results of the analyses indicate that significant differences were not observed between URM and non-URM participants. See Table 3 for results.

**Table 3:** Comparison on URM and non-URM Motivation to participate in the summer research program

I want to do research to:	URM			Non-URM			
	n	Mean	SD	n	Mean	SD	*p*
Explore my interest in science	144	4.53	0.78	159	4.58	0.75	0.515
Gain hands-on experience in research	144	4.73	0.63	160	4.78	0.63	0.528
Clarify which field I want to study	144	4.34	0.90	159	4.28	0.74	0.587
Clarify whether graduate school would be a good choice for me	144	3.72	1.09	160	3.71	1.12	0.940
Clarify whether I wanted to pursue a science research career	144	4.13	1.00	160	4.02	1.12	0.352
Work more closely with a particular faculty member	144	3.41	1.11	160	3.65	1.17	0.068
Participate in a program with a strong reputation	143	3.99	0.91	159	4.17	0.86	0.072
Get good letters of recommendation	144	3.99	0.97	160	4.08	0.96	0.421
Enhance my resume	144	4.18	0.97	159	4.30	0.89	0.281
Have a good intellectual challenge	144	4.63	0.64	160	4.72	0.64	0.208

### Students’ Prior Research Experience

The majority (56.8%) of participants indicated that they did not have any prior research experience. To examine differences between URM and non-URM participants, the frequencies and percentages were descriptively compared. The results are shown in Table 4. In general, a slightly larger percentage of URM participants report not having prior research experience (29.7%) compared to non-URM participants (27.4%). Among those with prior research experience, a slightly larger percentage of URM participants report one prior summer research program (10.9%) compared to non-URM (8.9%) participants. Other notable differences indicate a slightly larger percentage of non-URM participants reporting prior years and several summers of research experience (4.3%) than URM participants (1%). Relatedly, a slightly larger percentage of non-URM participants reported multiple academic semesters of research experience (5.0%) than URM participants (1.3%).

**Table 4:** Participants’ prior research experience by URM/non-URM status

	URM	Non-URM	
	% (n)	% (n)	*N*
No prior research experience	29.7 (90)	27.4 (83)	173
One summer research program experience	10.9 (33)	8.9 (27)	60
One academic semester of research experience	3.3 (10)	5.0 (15)	25
One academic semester and a summer research experience	1.3 (4)	2.3 (7)	11
Multiple academic semesters of research experience	1.3 (4)	5.0 (15)	19
Multiple academic semesters of research experience and several summer research programs	1.0 (3)	4.3 (13)	16

*Note* URM refer to African American/Black, Hispanic/Latino, and American Indian or Alaska Native (NSF, 2019); Missing data (n = 12)

### Research-Related Abilities, Skills and Learning Gains

Participants responded to 20 identical items before and after the SSRP that assessed research-related abilities, skills and learning gains. Together the 20 items demonstrated high internal consistency for the pre-program survey (20 items; *α* = 0.90), the post-program survey (20 items; *α* = 0.91), and was comparable to the SURE survey (20 items; *α* = 0.92; Lopatto, 2004). To examine whether responses differed between pre-and post-program assessments, paired sample *t*-tests were conducted (with Holm-adjusted *p* values). Based on the face validity of the items, the 20 research-related abilities, skills and learning gains items were grouped thematically. The results are shown in Table 5.

### Understanding the Scientific Process

Understanding the scientific process were assessed with five items: understanding how knowledge is constructed; understanding that scientific assertions require supporting evidence; understanding the research process in their field; understanding how scientists think; and understanding how to interpret results. Overall, results show that participants tended to report higher mean ratings (i.e., level of agreement with statements) at post-program than pre-program. Paired-sample *t*-tests results revealed statistically significant mean differences for understanding how knowledge is constructed (*t*(273) = 6.772, *p* < .001, *d* = 0.41), understanding that scientific assertions require supporting evidence (*t*(273) = 2.903, *p* < .01, *d* = 0.18), understanding of the research process (*t*(268) = 11.419, *p* < .001, *d* = 0.70), understanding how scientists think (*t*(273) = 8.689, *p* < .001, *d* = 0.53), and understanding how to interpret results (*t*(273) = 3.723, *p* < .001, *d* = 0.23).

### Research Skills and Abilities

Research skills and abilities were assessed with six items: learning ethical conduct; learning laboratory techniques; ability to work independently; ability to analyze data; understanding primary literature; and skill in science writing. Overall, results show that participants tended to report higher mean ratings (i.e., level of agreement with statements) at post-program than pre-program. With the exception of learning laboratory techniques (*t*(273) = 1.124, *p* = .26, *d* = 0.07), paired-sample *t*-tests results revealed statistically significant mean differences for: learning ethical conduct (*t*(273) = 4.868, *p* < .001, *d* = 0.29), ability to work independently (*t*(273) = 2.134, *p* < .05, *d* = 0.16), ability to analyze data and other information (*t*(273) = 5.387, *p* < .001, *d* = 0.33), ability to read and understand primary literature (*t*(273) = 4.550, *p* < .001, *d* = 0.28), and skill in science writing (*t*(273) = 9.405, *p* < .001, *d* = 0.57).

### Science/Research Identity

Science identity was assessed with four items: understanding science; becoming part of a learning community; tolerance for obstacles faced in the research process; and self-confidence. Overall, results show that participants tended to report higher mean ratings (i.e., level of agreement with statements) at post-program than pre-program. With the exception of understanding science (*t*(273) = 1.333, *p* = .18, *d* = 0.08), paired-sample *t*-tests results revealed statistically significant mean differences for: being a part of a learning community (*t*(272) = 2.717, *p* < .01, *d* = 0.16), having tolerance for obstacles faced in the research process (*t*(273) = 5.557, *p* < .001, *d* = 0.34), and having self-confidence (*t*(272) = 6.263, *p* < .001, *d* = 0.38).

### Applied Science Skills

Application of science skills was assessed with three items: understanding how scientists work on real problems; ability to integrate theory and practice; and knowing how to give an effective scientific oral and poster presentation. Overall, results show that participants tended to report higher mean ratings (i.e., level of agreement with statements) at post-program than pre-program levels. Paired-sample *t*-tests results revealed statistically significant mean differences for: understanding how scientists work on real problems (*t*(273) = 10.234, *p* < .001, *d* = 0.62), ability to integrate theory and practice (*t*(273) = 7.245, *p* < .001, *d* = 0.44), and knowing how to give an effective presentation (*t*(273) = 5.956, *p* < .001, *d* = 0.36).

### Readiness for Research Career

Readiness for a research career was assessed with two items: clarification of career path; and readiness for more demanding research. Results show that participants tended to report higher mean ratings (i.e., level of agreement with statements) at post-program than pre-program. Paired-sample *t*-tests results revealed statistically significant mean difference for readiness for more demanding research (*t*(273) = 4.953, *p* < .001, *d* = 0.30).

**Table 5:** Participants’ changes in research-related abilities, skills and learning gains from pre- to post-program

		Pre		Post			
Items	n	Mean	SD	Mean	SD	*p*	*d*
*Understanding the scientific process*							
Understanding how knowledge is constructed	274	3.71	0.80	4.05	0.70	< 0.001	0.41
Understanding that scientific assertions require supporting evidence	274	4.35	0.73	4.49	0.59	< 0.01	0.18
Understanding of the research process	269	3.43	0.87	4.10	0.68	< 0.001	0.70
Understanding how scientists think	274	3.49	0.82	3.96	0.73	< 0.001	0.53
Skill in the interpretation of results	274	3.79	0.72	3.97	0.63	< 0.001	0.23
*Research skills and abilities*							
Learning ethical conduct	274	3.95	0.84	4.23	0.77	< 0.001	0.29
Learning laboratory techniques	274	3.79	0.96	3.87	1.22	0.262	0.07
Ability to analyze data and other information	274	3.94	0.75	4.21	0.65	< 0.001	0.33
Ability to read and understand primary literature	274	3.88	0.82	4.14	0.72	< 0.001	0.28
Skill in science writing	274	3.23	0.97	3.88	0.80	< 0.001	0.57
*Science/research identity*							
Understanding science	274	3.87	0.75	3.93	0.82	0.184	0.08
Becoming part of the learning community	274	4.35	0.65	4.47	0.62	< 0.01	0.16
Tolerance for obstacles faced in the research process	274	4.00	0.71	4.25	0.65	< 0.001	0.34
Self-confidence	274	3.78	0.96	4.12	0.80	< 0.001	0.38
*Applied science skills*							
Skill in how to give an effective oral presentation	274	3.65	1.0	4.05	0.83	< 0.001	0.36
Understanding how scientists work on real problems	274	3.62	0.87	4.22	0.65	< 0.001	0.62
Ability to integrate theory and practice	274	3.53	0.79	3.95	0.74	< 0.001	0.44
*Readiness for research career*							
Clarification of career path	274	3.54	1.04	3.64	1.02	0.114	0.10
Readiness for more demanding research	274	3.81	0.85	4.08	0.84	< 0.001	0.30

### Comparison of URM and Non-URM Participant Post-Program Gains

A 2 (URM/non-URM) x 20 (post-program outcomes) MANOVA was conducted to compare URM and non-URM participants on all post-program research-related abilities, skills and gains items. The multivariate result was significant for URM and non-URM status, Wilk’s Lambda = 0.87, *F*(20, 251) = 1.902, *p* = .013, *η*_*p*_^*2*^ = 0.13, indicating a difference in the post-program means between URM and non-URM participants. Follow-up ANOVAs were conducted on the 20 post-program research-related abilities, skills and gains items. The results indicated non-significant differences (with Holm-adjusted p-values) between URM and non-URM participants for 15 out of the 20 *F* tests. Non-URM participants reported significantly higher means than URM participants on the following; learning laboratory techniques (*p* = .001); ability to integrate theory and practice (*p* = .002); readiness for more demanding research (*p* = .011); ability to analyze data and other information (*p* = .013); and understand how scientists work on real problems (*p* = .048). Descriptive data are presented in Table 6.

**Table 6:** MANOVA tests of between-subjects descriptive statistics of URM and non-URM responses on 20 post-program research-related abilities, skills and gains items

	URM			Non-URM				
Items	n	Mean	SD	n	Mean	SD	*F*	*p*
*Understanding the scientific process*								
Understanding how knowledge is constructed	130	4.02	0.68	141	4.11	0.71	1.345	0.247
Understanding that scientific assertions require supporting evidence	130	4.45	0.61	141	4.55	0.55	2.291	0.131
Understanding of the research process	130	4.08	0.70	141	4.16	0.63	1.141	0.286
Understanding how scientists think	130	3.90	0.71	141	4.04	0.74	2.343	0.127
Skill in the interpretation of results	130	3.91	0.67	141	4.05	0.59	3.474	0.063
*Research Skills and Abilities*								
Learning ethical conduct	130	4.26	0.82	141	4.21	0.71	0.359	0.550
Learning laboratory techniques	130	3.62	1.31	141	4.12	1.09	11.622	0.001***
Ability to work independently	130	4.25	0.71	141	4.35	0.61	1.369	0.243
Ability to analyze data and other information	130	4.12	0.69	141	4.32	0.59	6.320	0.013*
Ability to read and understand primary literature	130	4.08	0.71	141	4.21	0.73	2.170	0.142
Skill in science writing	130	3.89	0.70	141	3.89	0.89	0.000	0.989
*Science/Research Identity*								
Understanding science	130	3.92	0.74	141	3.95	0.89	0.074	0.785
Becoming part of the learning community	130	4.42	0.59	141	4.53	0.63	2.454	0.118
Tolerance for obstacles faced in the research process	130	4.19	0.71	141	4.33	0.58	2.935	0.088
Self-confidence	130	4.10	0.77	141	4.18	0.82	0.639	0.425
*Applied Science Skills*								
Skill in how to give an effective oral presentation	130	3.96	0.86	141	4.14	0.82	3.147	0.077
Understanding how scientists work on real problems	130	4.15	0.65	141	4.30	0.67	3.952	0.048*
Ability to integrate theory and practice	130	3.82	0.70	141	4.09	0.74	10.003	0.002**
*Readiness for Research Career*								
Clarification of career path	130	3.58	1.0	141	3.72	1.0	1.436	0.232
Readiness for more demanding research	130	3.96	0.89	141	4.22	0.78	6.493	0.011*

*Note*: *p < .05, **p < .01, ***p < .001

To determine how the dependent variables interact, the MANOVA was followed up with discriminant analysis. The results indicated a significant discriminant function, Λ = 0.872, *χ*2(20) = 35.531, *p* = .017 which explained 12.8% of the variance in the grouping variable (canonical *R*2 = 0.358, *η*_*p*_^*2*^ = 0.13). The correlations between the 20 post-program research-related abilities, skills and gains items and the discriminant function revealed moderate correlations for 2 items: learned laboratory techniques (*r* = .61) and ability to integrate theory and practice (*r* = .58). When examining the structure coefficients, both learned laboratory techniques (*r* = .54) and ability to integrate theory and practice (*r* = .50) had the strongest correlations. Overall, the one discriminant function was able to predict 64.9% group membership (URM and non-URM), with the non-URM group classified correctly (73.8%) more often than the URM group (55.4%).

### Impact of the Summer Research Program

The post-survey included a few questions that assessed the impact of the summer research experience. These items evaluated whether participants would choose to participate in future research opportunities, whether they would recommend the program to a friend or student (i.e., yes or no), and an open-ended question. Among the 291 participants who responded whether they would pursue future research endeavors, 87% indicated that they were “very likely” or “likely” to choose another research experience (see Table 7). Among 253 responses, 98.8% reported that they would recommend the program to a friend or student.

**Table 7:** Participants’ likelihood of pursuing future research opportunities by URM/non-URM status at post-program

	URM	Non-URM
	% (n)	% (n)
I will not choose to have another research experience	1.4 (4)	1.0 (3)
I am unlikely to choose another research experience	2.4 (7)	2.7 (8)
I am likely to choose another research experience	11.7 (34)	14.8 (43)
I am very likely to choose another research experience	27.5 (80)	32.6 (95)
Not applicable	3.1 (9)	1.4 (4)

*Note* Missing data (n = 24)

Four themes were identified from 224 responses. Some comments were more complex than others and were coded in multiple themes. Therefore, the overall percentage of comments per theme will not add to one hundred. We provide descriptions of each of the four themes, summaries of the data, and exemplars in the following section. The themes were (1) clarification of career or career path, (2) benefits associated with conducting hands-on research, (3) mentoring and professional development, and (4) skills and knowledge.

### Benefits Associated with Conducting Hands-on Research

About 39% of the comments were coded in the benefits associated with conducting hands-on research theme. Across these comments, participants noted the importance of conducting hands-on research. Specifically, the participants made several connections to the benefits of conducting research to further exposure to careers in research, immersion in the lab/research experience, increase in understanding how scientists or clinicians work, increase in soft and hard skills, increase in professional networks, and the importance of applied or translational research. For example, a student wrote:

This program allows for students to really apply their knowledge inside the classroom to a very different experience. Students find themselves in a situation where people actually questions what you have studied in class and really apply those concepts to make a difference in the world. In addition, you feel a sense of unity once you enter this program. You find yourself embedded within the research made in the laboratory that will one day impact you or a loved one. Throughout the summer, we were confronted with various studies being done. If I were to read these up online, they would seem daunting, almost unapproachable. But this program allowed me to gain my confidence and really throw myself into a field that I intend to pursue in the future. Overall, this experience opened new doors for my future and allowed me to be more confident in pursuing science. It was a fantastic experience! (17, Female, Asian, Cohort 3)

Some of the high school participants recognized the importance of early exposure to research. These remarks conveyed a sense of appreciation for the chances provided and an understanding that such summer research opportunities were not typically available to high school students. For example, a high school student wrote:

This program gave me opportunities that very few high school kids get to experience these days. I have seen how research is conducted in the real world and I have been able to confidently say that I have conducted my own research project and had real world results. This program is fantastic and I have very thankful for all of the work everyone puts in to make it possible. I would recommend this program to any of my friends who had any slight bit of interest in a scientific field. Thank you! (17, Female, Latina, Cohort 4)

### Mentoring and Professional Development

About 21% of the comments were coded in the mentoring and professional development theme. The respondents noted overall positive interactions with mentors, with some indicating meaningful experiences within the mentee-mentor relationship. For example, a college student wrote:

I have truly enjoyed working with my mentor who has really nurtured my love for health care and helped to solidify my future career goals. Being immersed in research all summer long has given me the skills I need to further my education in science and public health and has expanded my mind as to the impact that research and science can accomplish. (18, Female, Asian, Cohort 4)

Most of the participants noted that the positive interactions with mentors was associated to other factors such as further clarification of a career or academic path in STEM fields or the biomedical sciences, increase in understanding how scientists or clinicians work, increase in both soft (e.g., professional development, tolerance for obstacles) and hard skills (e.g., wet lab techniques), and increase in professional networks. For example, a participant wrote:

This program FAR exceeded my expectations, and gave me a hands-on opportunity to work closely with some of the best minds in the cancer research field. I learned how to handle failure and unexpected obstacles, as well as how to construct a plan of action/experimentation in response to a specific hypothesis or scientific observation. This experience is something that I will undoubtedly take with me the rest of my life–I couldn’t have imagined it going any better, and I can’t wait to recommend others to the program! (17, Female, White, Cohort 2)

Many of the comments focused on the mentor’s behaviors and actions. In general, participants reported that mentors provided a nurturing and supportive environment. They indicated that mentors were helpful, knowledgeable, and invested in the success and professional development of the participants. Some participants noted specific behaviors that were received well by the mentee. These behaviors included mentors frequently meeting with the participant, discussing academic and career paths, providing guidance in the research process, and providing hands-on research training. For example, a high school senior wrote:

My mentor was also an excellent source of information regarding his career, so he gave me a lot of advice that didn’t only deal with my project, which was very helpful for me in deciding my future. I had a fantastic experience and I believe my peers would as well. (17, Female, Asian, Cohort 3)

A small number (about 2%) of participants noted ways in which mentors could improve. These participants reported that they would have benefitted more from the mentee-mentor relationship if they had more frequent meetings with their mentor. In addition, they reported challenges due to the mentor not preparing work in the early stages of the program or not having enough time to devote to mentorship.

### Clarification of Career or Career path

About 21% of the comments were coded in the clarification of career theme. The respondents noted that participating in the SSRP was an opportunity to gain further clarification of a career or academic path in the biomedical sciences and STEM fields. Many participants conveyed that learning, conducting research, and interacting with mentors, students and staff helped them clarify their existing interests in science or STEM/biomedical careers. A participant wrote:

This program through seminars and interactions with my mentors, students, and staff truly cemented my interest in medicine, and I would like others to share the amazing experience I was blessed to have. (16, Male, African American, Cohort 7).

### Increased Skills and Knowledge

About 16% of the comments were coded in the increased skills and knowledge theme. In general, participants indicated that they learned a lot as a result of participating in the program. The learning gains were reported in the areas of research skills (e.g., laboratory skills), science knowledge (e.g., science topics in seminars), understanding the research process (e.g., “ups and downs” of daily research experiences), and professional development (e.g., presentation skills, scientific writing), For example, a participant wrote:

This was a very immersive science program. I learned much more than I expected, not just about the science and lab work, but also about scientific writing, presenting, responsibility, etc. I am much more confident in myself and my abilities than I was at the beginning of the program and feel very prepared for my college and beyond. (17, Female, Middle Eastern, Cohort 7)

Many participants noted gains in soft skills (e.g., persistence, work ethic, responsibility, independence, self-efficacy) as a result of participating in the program. For example, a high school junior noted:

[This program] provides a good challenge, allows us to interact with highly intelligent and successful individuals in the science field, learn more about the scientific research process, learn to take on long-term projects, learn how to be independent and find our own way, and learn how to ask for help. (15, Female, Native American, Cohort 6)

## Discussion

The Summer Student Research Program at UCSF BCH-Oakland was effective in producing positive academic outcomes for both URM and non-URM students. Student participants in the 9-week program experienced increases in research skills and abilities and post-baccalaureate aspirations. Findings from this study suggest that summer research programs may be a promising practice for increasing research skills and academic preparedness for students with STEM majors and research interests. Furthermore, the program’s inclusive approach underscores the potential for such initiatives to contribute significantly towards reducing disparities in STEM education and fostering a more diverse and robust scientific community.

Students who participated in the SSRP reported gains on 20 of the 20 items that assessed understanding the scientific process, research skills, science identity, applied science skills and readiness for a research career. Significant gains, a difference between pre- and post-survey responses, were observed in 18 of the 20 areas assessed (with several medium to large effect sizes). This suggests that the program was highly effective in improving student learning outcomes for diverse learners.

Students experienced significant gains in all five understanding the scientific process areas and all applied science skills that were measured. Students experienced significant gains in five out of six research skill and ability domains measured, with the exception of laboratory techniques. It is worth noting that non-URM students reported higher post-program gains in the laboratory techniques area than URM-students. This finding may be attributed to differences in the type of research conducted during the SSRP (e.g., clinical or laboratory) or related to students’ prior research experiences. Students who were conducting a clinical research project would likely not report gains in laboratory skill development. It is also conceivable that students with more prior lab experience would continue to develop research gains, while others lacking similar experience might have different outcomes with early lab experiences. Future research should examine best practices for cultivating the laboratory skills of diverse learners.

Some of the largest gains for students, were in the areas of understanding of the research process, understanding how scientists think, developing skills in science writing and understanding how scientists work on real problems. These findings are important because these skills are critical to undergraduate degree attainment, and may positively influence academic persistence. Additionally, these findings suggest that the program is building students’ capacity for academic and professional work in a range of STEM, STEM-adjacent, and interdisciplinary fields. As the biomedical and STEM fields continue to evolve and have increasing overlap with areas including technology, communication, and business, the development of a range of interdisciplinary skills has significant benefits for students and for society.

Another positive outcome of the program is that it produced changes in terminal degree intention among some participants. In comparing pre- and post-program survey data, some students reported an increased interest in all postgraduate degree pursuits, including desires to pursue a Master of Arts in a biomedical or STEM related field, a Ph.D. in biomedicine or STEM, or an M.D. Post-program survey data showed a decrease in interest in pursuing a joint M.D./Ph.D. program. This decrease is possibly related to the fact that the SSRP produced increased knowledge in the processes of postgraduate application and medical research, and likely clarified assumptions and misconceptions around requirements for pursuing both degrees. It is likely that student’s expectations adjusted with the new information and increased clarity around academic and professional pathways.

Findings from this program point to the potential of a well-structured intensive program to improve outcomes for URM-students and other diverse learners. Post-survey data indicated that 87% of program participants were “very likely” or “likely” to choose another research experience, with no differences reported between URM and non-URM students. Students of all backgrounds experienced overlap in other areas as well. The summer research program’s ability to increase students’ self-efficacy across the board in academics and career preparedness points to the strengths of this particular program, as well as the model overall as a promising practice.

Finally, a key finding of this work is its’ practical impact. This summer research program produced positive long-term post-program outcomes for participants. Students reported a range of improvements in knowledge and research skills. However, improvement in research readiness, understanding the scientific process, and research readiness have significant practical implications, as they are all core areas that point to these students’ present and future readiness to work in STEM and other related fields.

This study was not without limitations. There is perhaps some selection bias in the sample, as students enrolled in the SSRP were very motivated to participate in the program, and were selected through a highly competitive application process. Students were however, similar in ethnic, education and cultural background to the U.S. statistics of URM youth. Moreover, the selection committee uses a holistic application process, focusing more on the students’ curiosity for science and not just high academic achievers. Emphasis is also made at recruiting from local community colleges and state, to ensure students are diverse academically, economically, socially and culturally. The research design attempted to address these limitations as well. The pre-post within subjects design has several benefits such as higher statistical power to detect meaningful differences, allows examination of change over time, and minimizes the influence of extraneous variables. Additionally, the large dataset which included data across an eight-year span and the high response rates to both survey administrations increased the internal validity of the study and generalizability of the findings.

On the whole, this study highlights the impact of one innovative practice in higher education – the short-term summer research program. While short term research programs offer universities the opportunity to provide intensive training to students in a short period of time, thus saving time and money, there is a lack of rigorous studies and empirical data about their impact. In this study, statistical analyses demonstrated that a 9-week, intensive program that utilized supports consistent with Graham et al.’s persistence framework (2013), including early research experiences, active learning, and membership and a learning community, could produce positive academic outcomes for undergraduate STEM students. By focusing on the summer intensive format, this study identifies a promising practice for higher education.

Another core finding of this study is that such programs, which provide high levels of student engagement and research support, can generate positive learning outcomes for both URM and non-URM undergraduate STEM students. The gains in academic outcomes and the similarity of outcomes between URM and non-URM highlight the potential of such programs in increasing students’ research skills and post-baccalaureate aspirations for diverse learners, which in turn can increase the diversity in biomedical sciences and health-related professions. Future research should explore long-term gains experienced by SSRP alumni and persistence in STEM majors and careers.
